# Effects of sodium-glucose cotransporter 2 inhibitors on bone metabolism in patients with type 2 diabetes mellitus: a systematic review and meta-analysis

**DOI:** 10.1186/s12902-024-01575-8

**Published:** 2024-04-24

**Authors:** Jing Wang, Xin Li, Yang Li, Chen Lei

**Affiliations:** 1 Office of Academic Research, General Hospital of Ningxia Medical University, 750004 Yinchuan, Ningxia China; 2 Department of Nutrition, General Hospital of Ningxia Medical University, 750004 Yinchuan, Ningxia China; 3 First Clinical Medical College, Ningxia Medical University, 750004 Yinchuan, Ningxia China; 4 Department of Geriatrics and Special Needs, General Hospital of Ningxia Medical University, No. 804 South Shengli Street, 750004 Yinchuan, Ningxia China

**Keywords:** Type 2 diabetes mellitus, Bone metabolism, Bone mineral density, SGLT2 inhibitor, Meta-analysis

## Abstract

**Background:**

Sodium glucose cotransporter 2 (SGLT2) inhibitors are widely used in type 2 diabetes mellitus (T2DM) therapy. The impact of SGLT2 inhibitors on bone metabolism has been widely taken into consideration. But there are controversial results in the study on the effect of SGLT2 inhibitors on bone metabolism in patients with T2DM. Therefore, we aimed to examine whether and to what extent SGLT2 inhibitors affect bone metabolism in patients with T2DM.

**Methods:**

A literature search of randomized controlled trials (RCTs) was conducted through PubMed, Web of Science, Embase, Cochrane databases, and Scopus from inception until 15 April 2023. Eligible RCTs compared the effects of SGLT2 inhibitors versus placebo on bone mineral density and bone metabolism in patients with T2DM. To evaluate the differences between groups, a meta-analysis was conducted using the random effects inverse-variance model by utilizing standardized mean differences (SMD).

**Results:**

Through screening, 25 articles were finally included, covering 22,828 patients. The results showed that, compared with placebo, SGLT2 inhibitors significantly increased parathyroid hormone (PTH, SMD = 0.13; 95%CI: 0.06, 0.20), and cross-linked C-terminal telopeptides of type I collagen (CTX, SMD = 0.11; 95%CI: 0.01, 0.21) in patients with T2DM, decreased serum alkaline phosphatase levels (ALP, SMD = -0.06; 95%CI: -0.10, -0.03), and had no significant effect on bone mineral density (BMD), procollagen type 1 N-terminal propeptide (P1NP), 25-hydroxy vitamin D, tartrate resistant acid phosphatase-5b (TRACP-5b) and osteocalcin.

**Conclusions:**

SGLT2 inhibitors may negatively affect bone metabolism by increasing serum PTH, CTX, and decreasing serum ALP. This conclusion needs to be verified by more studies due to the limited number and quality of included studies.

**Systematic review registration:**

PROSPERO, identifier CRD42023410701

**Supplementary Information:**

The online version contains supplementary material available at 10.1186/s12902-024-01575-8.

## Research in context

SGLT2 inhibitors have been widely used in clinical practice for their good cardiorenal protective and hypoglycemic effects. However, their effects on bones are still controversial. The drug has been shown to have a potential adverse effect on bone in multiple animal experiments. However, in the latest meta-analysis, it was not found that the risk of fracture increased in patients with type 2 diabetes mellitus (T2DM) treated with SGLT2 inhibitors.

Can SGLT2 inhibitors affect bone mineral density and bone metabolism in patients with T2DM?

We found that SGLT2 inhibitors may have a negative effect on bone in patients with T2DM.

When T2DM is treated in clinical work, doctors will pay more attention to the monitoring of bone safety. And we provided a reference for the use of SGLT2 inhibitors.

## Introduction

It is well known that type 2 diabetes mellitus (T2DM) is characterized by persistently elevated blood glucose or elevated postprandial blood glucose containing carbohydrates [1]. As a chronic non-communicable disease, its prevalence is increasing worldwide, especially related to the gradual entry of people into an aging society, high calorie intake, and a sedentary lifestyle [2]. Recent studies have shown that in addition to the cardiovascular, ocular, renal and neurological complications of the disease in patients, bone strength is also impaired and leads to an increased risk of fractures [3]. The presence of T2DM is associated with a prevalent metabolic disorder that has detrimental effects on bone metabolism, leading to an increased susceptibility to fractures [4, 5]. Among the various types of osteoporotic fractures, individuals with T2DM face a heightened risk for hip fractures, which are considered the most severe, as well as limb fractures such as those occurring in the leg or ankle [6].

The anti-diabetic drugs currently applied clinically have certain effects on the bone metabolism of patients [7]. Sodium–glucose cotransporter 2 (SGLT2) inhibitor is one of the new hypoglycemic drugs. It can reduce glucose re-absorption by inhibiting SGLT2 in proximal tubules of the kidney, thus promoting urine glucose excretion and reducing blood glucose [8]. In recent years, studies on the effects of SGLT2 inhibitors on bone metabolism have been continuously released, and the existing relationship between the two is still controversial. Theoretically, SGLT2 inhibitors increase renal tubular reabsorption of phosphate and serum parathyroid hormone concentration [9].

Considering the significant economic and social burden caused by bone health issues and associated fracture risks, it is imperative to conduct a comprehensive evaluation of the impact of SGLT2 inhibitors on fractures and bone metabolism. In view of the fact that there are still controversial results in the study on the effect of SGLT2 inhibitors on bone metabolism in patients with T2DM, we conducted a systematic and comprehensive analysis of the existing research results in order to provide reference for the selection of SGLT2 inhibitors in the treatment of T2DM in clinical work.

## Methods

### Protocol and registration

The protocol of this systematic review and meta-analysis has been registered in PROSPERO (registration no. CRD42023410701).

### Eligibility criteria

We included randomized controlled trials (RCTs) comparing the efficacy of SGLT2 inhibitors versus placebo, in English only. Eligible participants were adults with T2DM, regardless of background hypoglycemic therapy. Interventions should last for at least 12 weeks and the outcomes should include at least one of bone mineral density or bone metabolism.

### Search strategy

We searched PubMed, Web of Science, Embase, Cochrane databases, and Scopus on 15 April 2023 for English-language studies. Detailed information about our search strategy was presented in the electronic supplementary material (Table S1). To avoid omitting any eligible studies, any terms related to “SGLT2 inhibitor” were searched.

### Selection process

All search results were downloaded into EndNote (version X9, Thomson Reuters, Philadelphia, PA, USA) to eliminate duplication. Two reviewers independently performed a preliminary screening of the title and abstract. Remaining articles were read through the full text to determine inclusion, and the reasons for excluded articles were recorded. Any disagreements were resolved by a third reviewer. Articles that could not get the required data were also excluded. Articles for which the required data were not available after contacting the corresponding author were also excluded.

### Data collection and risk of bias assessment

Data extraction was done by two independent reviewers and arbitrated by a third reviewer. The relevant information extracted from the included articles mainly included: (1) Basic information: first author, publication year, sample size, and the number of experimental and control groups. (2) Characteristics of research subjects: gender, age, glycated hemoglobin, BMI, SGLT2 inhibitor type and dose, and duration of treatment; (3) Outcomes: Mean ± standard deviation (SD) of post-treatment relative baseline changes in bone mineral density (BMD) and bone metabolism-related indicators including parathyroid hormone (PTH), cross-linked C-terminal telopeptides of type I collagen (CTX), alkaline phosphatase (ALP), 25-hydroxy vitamin D, procollagen type 1 N-terminal propeptide (P1NP), osteocalcin, and Tartrate resistant acid phosphatase-5b (TRACP-5b); (4) Relevant information described in the literature that can be used to assess the risk of bias.

The risk of bias will be assessed by two authors independently using the RoB2 tool for the included RCTs [10]. Using the RoB2 tool, we will assess domains such as randomization process, assignment and adhering to intervention, missing data and measurement of outcome, and finally categorize the studies as having a low, some concern, or high risk of bias.

### Statistical analysis

We will pool the results using a random-effects meta-analysis, using standard mean difference (SMD) for continuous outcomes, and calculate 95% confidence interval (CI). A *p*-value < 0.05 was considered statistically significant. The Chi-square test combined with I-value analysis was used to judge the heterogeneity among the articles. When the heterogeneity of the studies in each group was relatively large (*P* < 0.05, I^2^ ≥ 50%), the source of heterogeneity needed to be clarified. Subsequent subgroup analysis or sensitivity analysis was conducted to explain the reasons for heterogeneity. Egger’s tests were performed to assess publication bias. R (version 4.2.3) and the statistical package ‘meta’ were used for analysis.

## Results

### Search results

**Fig. 1:**
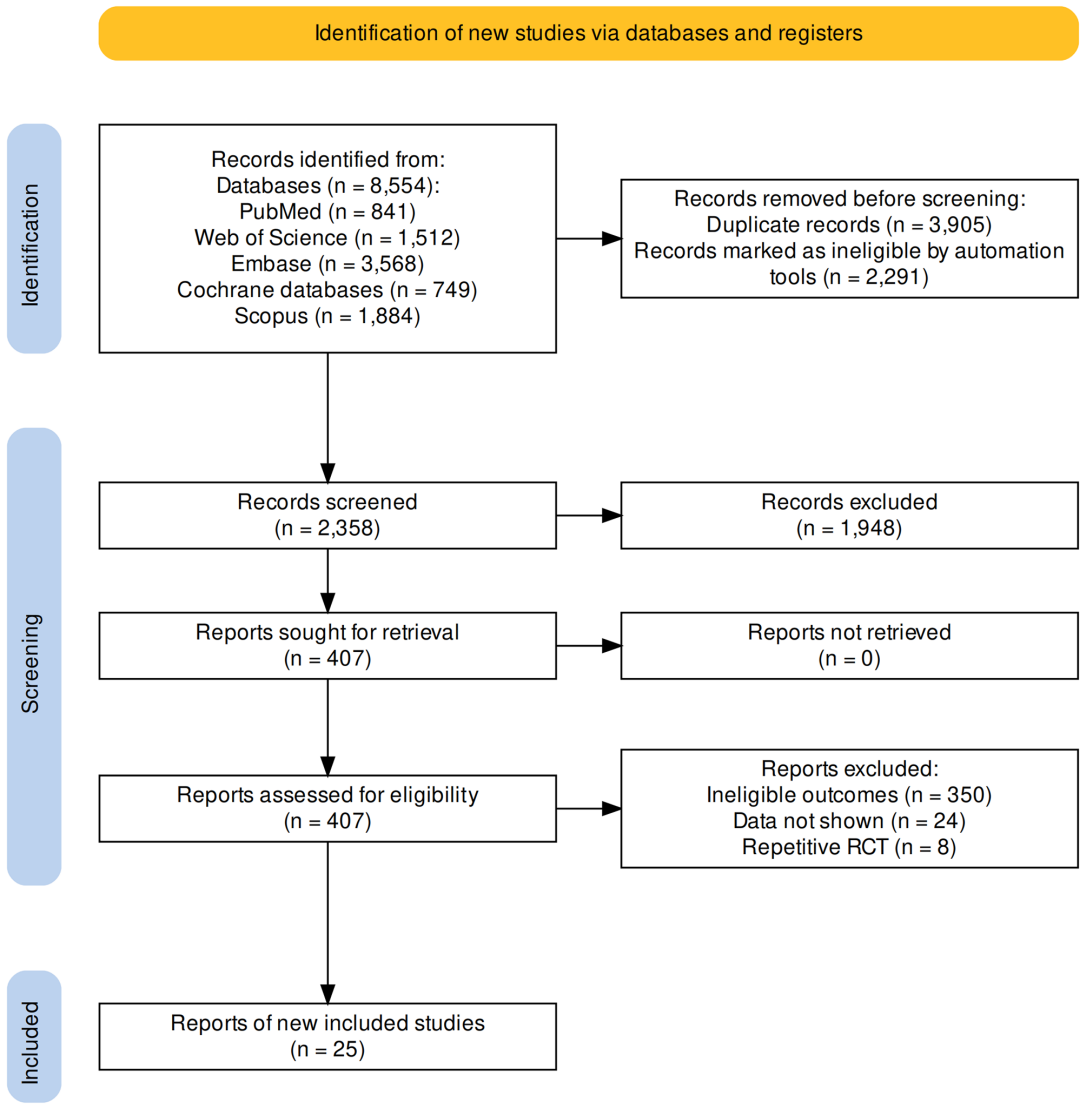
Flow diagram of the identification of eligible trials

According to the established retrieval strategy, we screened a total of 8554 studies from 5 databases. After a series of screenings, 25 studies ultimately met the eligibility criteria, totaling 22,828 unique participants. Twenty-three studies included in the analysis were RCTs [11–33], and two studies were for RCTs Pooled analysis [34, 35] (Fig. 1).

### Study characteristics

The study characteristics were summarized in Table 1. A total of 22,828 participants from 25 RCTs were randomly assigned to one of five SGLT2 inhibitors (canagliflozin, dapagliflozin, empagliflozin, ipragliflozin, and ertugliflozin) or placebo. Sample sizes for individual trials ranged from 40 to 12,620 participants, and the average trial duration was 55 weeks (range 12–104 weeks).

**Table 1:** Characteristics of included studies

Study	NCT number	SGLT2 inhibitor	Background therapy	Sample size	Male (%)	Mean Age (year)	Mean HbA1c (%)	Mean BMI (kg/m²)	Follow up (weeks)
Bolinder et al. (2014) [11]	NCT00855166	DAPA: 10 mg	MET	180	55.6	60.7	7.2	31.9	102
Bailey et al. (2014) [12]	NCT00528372	DAPA: 2.5 mg, 5 mg, 10 mg	Naive treatment	274	48.2	52.2	7.9	NR	102
Kohan et al. (2013) [13]	NCT00663260	DAPA: 5 mg, 10 mg	OAD	252	65.1	67.0	8.4	NR	104
Rosenstock et al. (2012) [14]	NCT00683878	DAPA: 5 mg, 10 mg	PIOG	420	49.5	53.5	8.4	NR	48
Wilding et al. (2012) [15]	NCT00673231	DAPA: 2.5 mg, 5 mg, 10 mg	INS ± OAD	807	47.8	59.3	8.5	33.1	48
Araki et al. (2017) [16]	NCT02157298	DAPA: 5 mg	INS ± OAD	182	70.9	58.1	8.4	26.6	16
Schumm et al. (2015) [17]	NCT01217892	DAPA: 2.5 mg, 5 mg, 10 mg	MET	400	44.9	57.7	7.8	32.6	16
Wilding et al. (2013) [18]	NCT01117584	IPRA: 50 mg	MET	134	50.7	58	7.7	31.5	12
Lu et al. (2016) [19]	NCT01505426	IPRA: 50 mg	MET	170	45.3	53	7.7	26.8	24
Han et al. (2018) [20]	NCT02452632	IPRA: 50 mg	MET + SIT	139	49.6	57.5	7.9	25.8	24
Fonseca et al. (2013) [21]	NCT01071850	IPRA: 50 mg	OAD	136	48.5	53	8	31.5	12
Min et al. (2017) [22]	NCT01505426	IPRA: 50 mg	MET	82	46.3	56.1	7.6	25.8	24
Kashiwagi et al. (2015) [23]	NCT01057628	IPRA: 50 mg	OAD	129	69.8	59.4	8.3	25.5	16
Gallo et al. (2019) [24]	NCT02033889	ERTU: 5 mg, 15 mg	GLIM	621	46.4	56.6	8.1	31.1	104
Ji et al. (2015) [25]	NCT01381900	CANA: 100 mg, 300 mg	MET ± SU	676	53.6	56.2	8	25.7	18
Bilezikian et al. (2016) [26]	NCT01106651	CANA: 100 mg, 300 mg	OAD	714	55.5	63.6	7.7	31.6	104
Yale et al. (2014) [27]	NCT01064414	CANA: 100 mg, 300 mg	SU or INS	269	60.6	68.5	8.0	33.0	52
Rosenstock et al. (2012) [28]	NCT00642278	CANA: 100 mg, 300 mg	MET	193	53.4	52.4	7.8	31.3	12
Rodbard et al. (2016) [29]	NR	CANA: 100 mg, 300 mg	MET + SIT	213	56.8	57.4	8.5	32.0	26
Sha et al. (2014) [30]	NCT01483781	CANA: 300 mg	OAD	36	86.1	62.8	7.7	29.8	12
Usiskin et al. (2014) [34]	NCT01081834 NCT01106625 NCT01106677 NCT01106690	CANA: 100 mg, 300 mg	OAD	2313	49.5	55.9	8	32.1	26
Sone et al. (2020) [31]	NCT02589639	EMPA: 10 mg, 25 mg	INS ± OAD	269	72.6	58.7	8.8	26.9	16
Kohler et al. (2018) [35]	NCT01289990 NCT01210001NCT01177813NCT01164501NCT00789035NCT00749190NCT0088511 NCT01011868NCT01947855NCT01193218NCT01370005NCT01306214; NCT01131676	EMPA: 10 mg, 25 mg	OAD	12,620	64.8	60.6	8.1	30.4	78
Rau et al. (2022) [32]	EudraCT: 2016-000172-19	EMPA: 10 mg	OAD	42	81.0	62.0	7.7	31.3	3month
Kullmann et al. (2022) [33]	NCT03227484	EMPA: 25 mg	OAD	40	40.0	59.9	5.7	31.5	8

BMI: body mass index; DAPA: dapagliflozin; IPRA: Ipragliflozin; ERTU: ertugliflozin; CANA: canagliflozin; EMPA: empagliflozin; MET: metformin; OAD: oral antidiabetic drugs; PIOG: pioglitazone; INS: insulin; SIT: sitagliptin; GLIM: Glimepiride; SU: sulfonylureas; NR: not reported

**Fig. 2:**
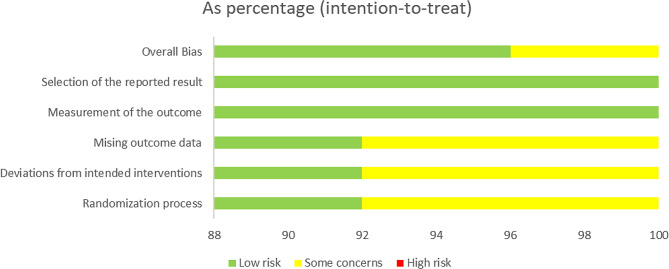
Risk of bias assessments of included studies

The risk of bias in the 25 RCTs is summarized in Fig. 2. Most of the trials included in the meta-analysis were judged to have a low risk of bias.

### Meta-analysis results

#### Bone mineral density

**Fig. 3:**
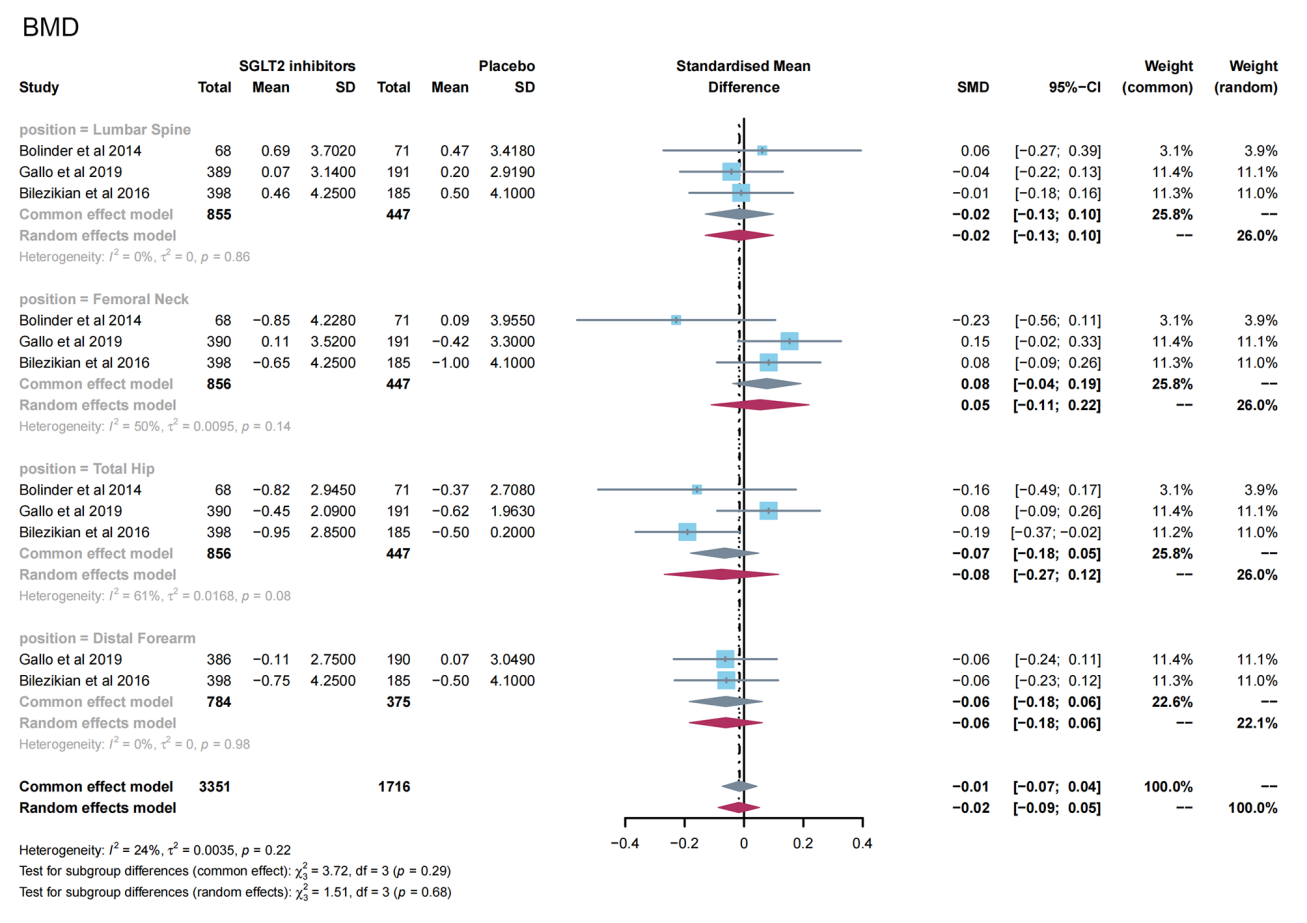
Meta-analysis of the effect of Sodium–glucose cotransporter 2 (SGLT2) inhibitors on BMD compared with placebo. BMD, bone mineral density

A total of 3 studies [11, 24, 26] reported the effects of SGLT2 inhibitors on BMD in patients with T2DM. The results of the overall and subgroup meta-analysis are presented in Fig. 3. There was no significant difference in BMD after treatment between the SGLT2 inhibitor group and the placebo group (SMD = -0.02; 95%CI: -0.09, 0.05). In subgroup analyses of bone sites, there was also no significant change in BMD in the two groups (lumbar spine, SMD = − 0.02, 95%CI: −0.13, 0.10; femoral neck, SMD = 0.05, 95%CI: −0.11, 0.22; total hip, SMD = -0.08, 95%CI: −0.27, 0.12; and distal forearm, SMD = − 0.06, 95%CI: −0.18, 0.06). No evidence of publication bias was observed (Table S2).

#### Bone metabolism

**Fig. 4:**
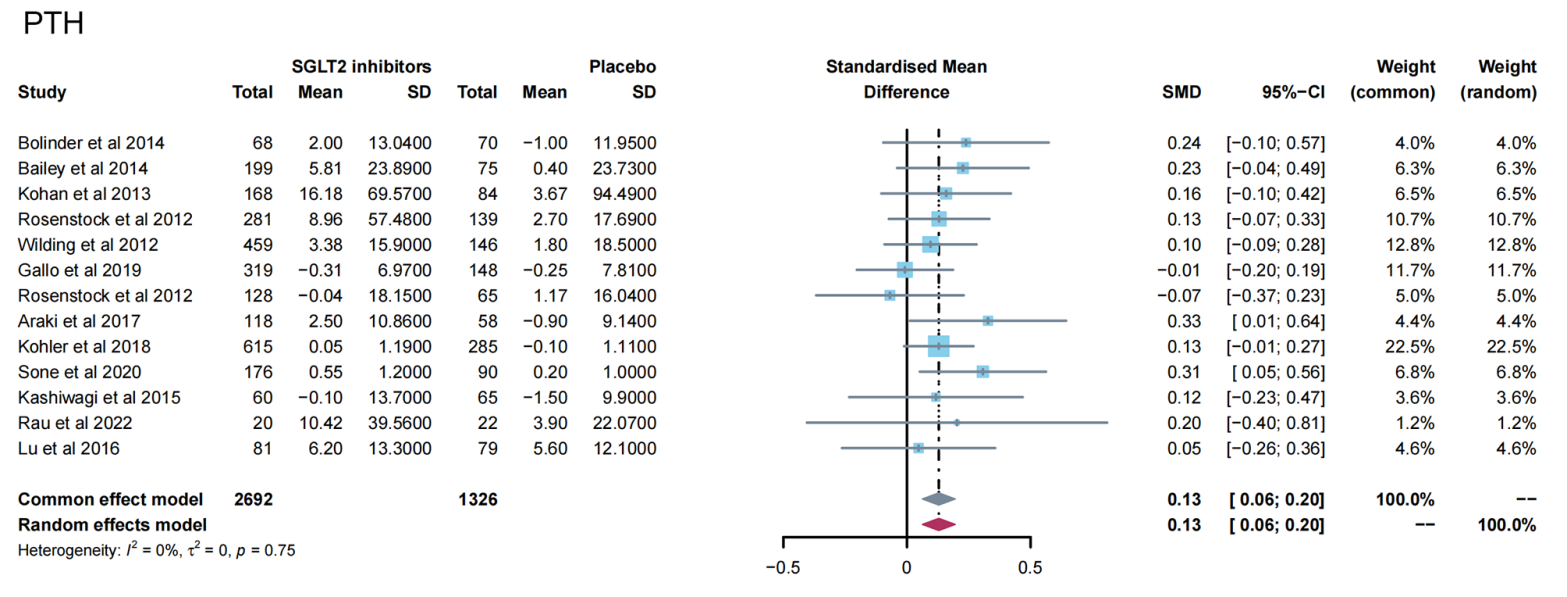
Meta-analysis of the effect of Sodium–glucose cotransporter 2 (SGLT2) inhibitors on PTH compared with placebo. PTH, parathyroid hormone

**Fig. 5:**
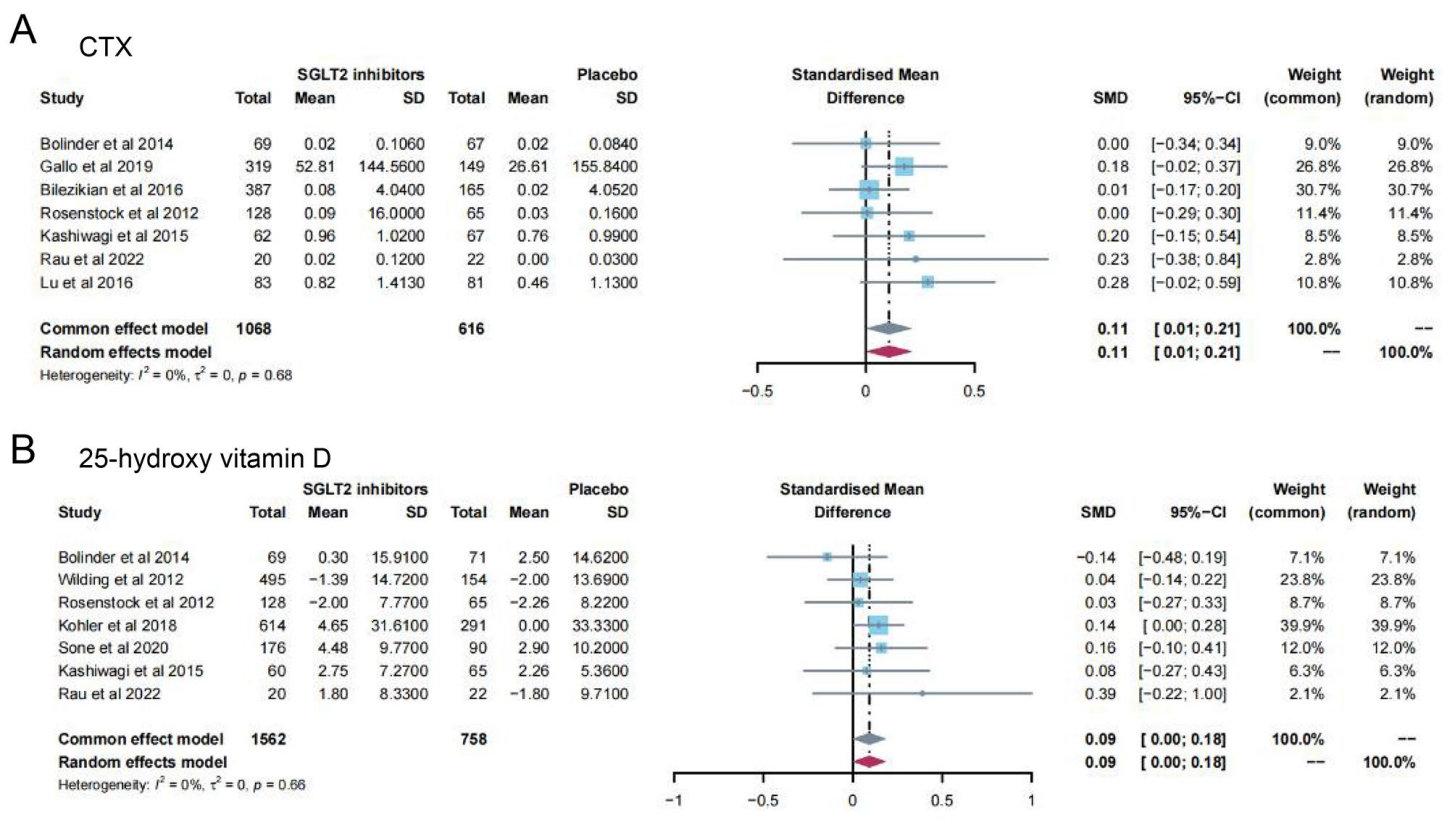
Meta-analysis of the effect of Sodium–glucose cotransporter 2 (SGLT2) inhibitors on CTX (**A**) and 25-hydroxy vitamin D (**B**) compared with placebo. CTX, Cross-linked C-terminal telopeptides of type I collagen

**Fig. 6:**
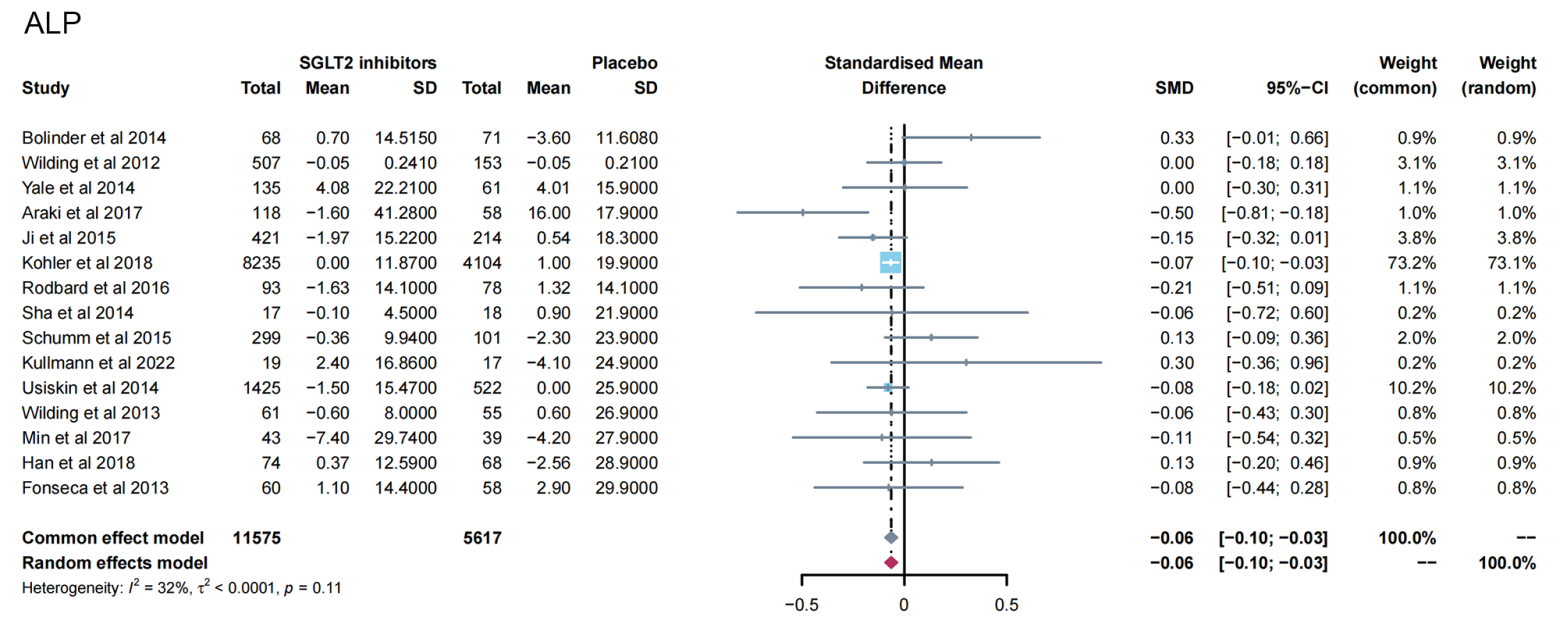
Meta-analysis of the effect of Sodium–glucose cotransporter 2 (SGLT2) inhibitors on ALP compared with placebo. ALP, Alkaline phosphatase

**Fig. 7:**
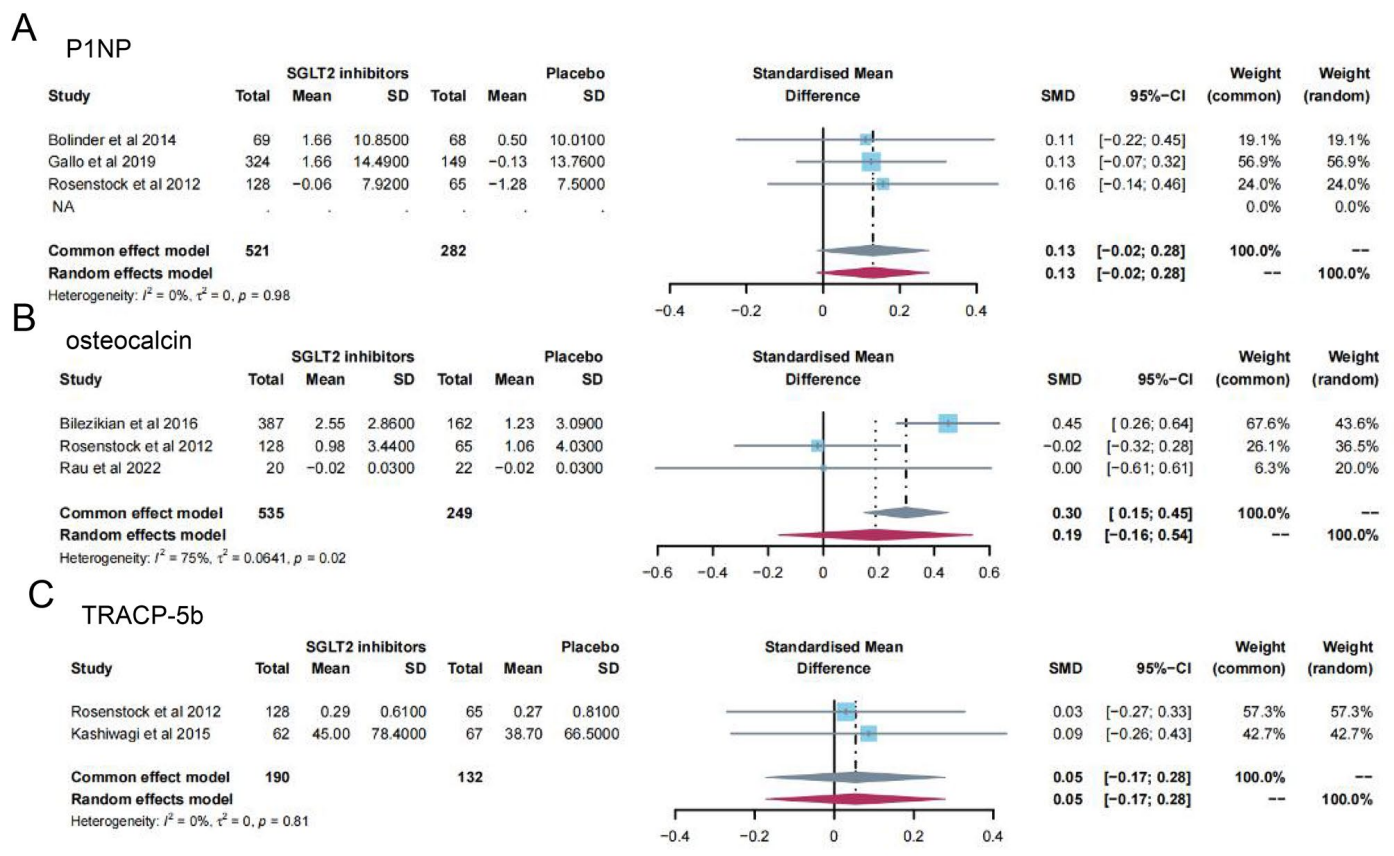
Meta-analysis of the effect of Sodium–glucose cotransporter 2 (SGLT2) inhibitors on P1NP (**A**), osteocalcin (**B**) and TRACP-5b (**C**) compared with placebo. P1NP, Procollagen type 1 N-terminal propeptide; TRACP-5b, Tartrate resistant acid phosphatase-5b

13 studies [11–16, 19, 23, 24, 28, 31, 32, 35] reported PTH levels after SGLT2 inhibitor treatment (Fig. 4). 7 papers compared CTX [11, 19, 23, 24, 26, 28, 32] and 25-hydroxy vitamin D [11, 14, 15, 23, 31, 32, 35] levels after treatment (Fig. 5A-B). 15 papers [11, 15, 16, 18, 20–22, 25, 27, 29, 30, 34, 35] reported ALP levels after treatment (Fig. 6). 3 papers compared P1NP [11, 14, 24] and osteocalcin [14, 26, 32] levels after treatment (Fig. 7A-B). 2 papers [23, 28] reported TRACP-5b levels after treatment (Fig. 7C). Except for osteocalcin (*P* = 0.02, I^2^ = 75%), no significant heterogeneity was observed. Meta results showed that, compared with placebo, SGLT2 inhibitors significantly increased PTH levels (SMD = 0.13; 95%CI: 0.06, 0.20) and CTX levels (SMD = 0.11; 95%CI: 0.01, 0.21), while significantly decreased ALP levels (SMD = -0.06; 95%CI: -0.10, -0.03). However, there was no significant difference in 25-hydroxy vitamin D (SMD = 0.09; 95%CI: 0.00, 0.18), P1NP (SMD = 0.13; 95%CI: -0.02, 0.28), osteocalcin (SMD = 0.19; 95%CI: -0.16, 0.54), and TRACP-5b (SMD = 0.05; 95%CI: -0.17, 0.28) after treatment between the SGLT2 inhibitor group and the placebo group.

In addition, no evidence of publication bias was observed for any of the above outcomes (Table S2).

## Discussion

The combined detection of BMD and bone turnover markers can be used to evaluate bone metabolism in patients. However, the changes of bone turnover markers are more sensitive [36]. In this study, after a comprehensive literature search and analysis, 25 studies were finally included for meta-analysis. Our results suggested that SGLT2 inhibitors had no significant effect on BMD in patients with T2DM compared to placebo. However, due to the short follow-up period and limited number of the RCTs included in the studies, more long-term studies are needed to accurately determine the impact of SGLT2 inhibitors on BMD.

In terms of bone metabolism, we observed that SGLT2 inhibitors significantly increased serum PTH and CTX levels and decreased serum ALP levels in patients with T2DM. This presents a seemingly paradoxical situation, as it is traditionally understood that elevated levels of PTH normally stimulate bone formation, which in turn increases levels of ALP, the active marker of bone formation [37]. This reflects the discrepancy between increased PTH levels and decreased ALP levels in patients using SGLT2 inhibitors underscores the complexity of the drugs’ impact on bone metabolism. It suggests a multifactorial influence involving immediate metabolic changes, differential effects on bone remodeling phases, the intricate role of RAAS activation, and the body’s broader compensatory responses [38]. In addition, no statistically significant effect of SGLT2 inhibitors on P1NP, TRACP-5b, 25-hydroxy vitamin D, and osteocalcin was observed in this study. However, although CTX and ALP levels change significantly in the meta-analysis, no single report shows a significant increase in CTX and only one study found a significant reduction of ALP. The reason for these phenomena can be attributed to the short duration of the study. The studies included this time are up to just over 3 months (104 days). Current research suggests that short-term studies (3 months) may not sufficiently capture significant changes in bone metabolism markers due to the physiological lag between alterations in glucose metabolism and their impact on bone remodeling processes []. In contrast, studies extending beyond 6 to 12 months are considered more likely to demonstrate meaningful changes in these markers [37, 39]. Further research, particularly studies with longer follow-up periods and detailed analyses of bone quality and turnover markers, is needed to fully elucidate these relationships.

The exact mechanism of the negative effects of SGLT2 inhibitors on bone health remains unknown. A study has shown that SGLT2 is not expressed in either the osteoblast lineage or the osteoclast lineage [40]. SGLT1 was detected in MC3T3-E1 differentiated osteoblasts, but its expression level was low. Therefore, the effects of these drugs on bone may be indirect [41]. SGLT2 inhibitors destroy serum calcium, phosphate, and vitamin D homeostasis [42]. As reabsorption of sodium in the proximal renal tubules decreases, the activity of sodium-phosphate co-transporters at the apical membrane increases. Serum phosphate levels further increase, inducing parathyroid cells and osteoblasts to secrete PTH and fibroblast growth factor 23 (FGF23). PTH causes bone resorption. While FGF23 promotes urinary phosphate excretion, inhibition of 1-αhydroxylase causes a decrease in 1,25-dihydroxvitamin D levels [43]. The decrease in blood sodium concentration can also directly affect osteoclasts, leading to an increase in bone fragility [44]. In the opposite way, calcium is reabsorbed by sodium-calcium cotransporters. The inhibition of SGLT2 leads to increased excretion of urine glucose and urine calcium, and the decrease of serum calcium causes secondary hyperparathyroidism [9]. It has been verified that the main results in our study suggested SGLT2 inhibitors could significantly increase serum PTH. Unfortunately, there are no more clinical studies reporting the effects of SGLT2 inhibitors on FGF23 in patients with T2DM.

SGLT2 inhibitors provide modest weight loss. A reduction in mechanical pressure on the bone tissue may decrease bone density and enhance bone turnover [45]. This may partly explain the reduction in total hip bone density in T2DM patients with canagliflozin. Weight loss also decreases aromatase activity, resulting in decreased estradiol levels that severely affect bone density and bone turnover [46, 47]. In addition to the indirect effects of SGLT2 inhibitors on bone metabolism, adverse events associated with these agents due to osmotic diuresis and volume consumption (orthostatic hypotension, postural dizziness, etc.) may increase the risk of falls and fractures [48].

There are some limitations to consider in this study. Most studies containing SGLT2 inhibitors focused on the cardiorenal effects. The main outcomes did not include bone health or relevant data were not shown. Therefore, some types of SGLT2 inhibitors received few articles and participants. Important confounding factors such as diet, exercise level, and solar radiation were not reported in some original studies and cannot be corrected. Since T2DM requires a combination of drugs in most cases, the background treatment for each patient cannot be unified, and there may be other drugs that also affect bones, leading to error in the results.

## Conclusion

Although further studies are needed, the results of our study have demonstrated the possible negative effects of SGLT2 inhibitors on bone health in patients with T2DM. However, there is still a lack of human studies regarding the effects of SGLT2 inhibitors on bone microarchitectural changes in patients with T2DM. Further preclinical or clinical data are needed to elucidate the effects on bone matrix mineralization and collagen fiber distribution. SGLT2 inhibitors have a good hypoglycemic effect and cardiorenal protection, but they may have a secondary effect on bone turnover. The long-term safety of this effect on bones deserves continued monitoring as the use of this drug becomes more routine in patients with T2DM.

### Electronic supplementary material

Below is the link to the electronic supplementary material.

Supplementary Material 1

## Data Availability

Datasets used in this article are available from the corresponding author on reasonable request.

## Abbreviations

ALP: Alkaline phosphatase
BMD: Bone mineral density
CTX: Cross-linked C-terminal telopeptides of type I collagen
FGF23: Fibroblast growth factor 23
P1NP: Procollagen type 1 N-terminal propeptide
PTH: Parathyroid hormone
RCTs: Randomized controlled trials
SGLT2: Sodium–glucose cotransporter 2
SMD: Standard mean difference
TRACP-5b: Tartrate resistant acid phosphatase-5b

## References

[1] Vijan S (2019). Type 2 diabetes. Ann Intern Med.

[2] Picke AK, Campbell G, Napoli N, Hofbauer LC, Rauner M (2019). Update on the impact of type 2 diabetes mellitus on bone metabolism and material properties. Endocr Connections.

[3] Koromani F, Ghatan S, van Hoek M, Zillikens MC, Oei EHG, Rivadeneira F, Oei L (2021). Type 2 diabetes Mellitus and vertebral fracture risk. Curr Osteoporos Rep.

[4] Nilsson AG, Sundh D, Johansson L, Nilsson M, Mellstrom D, Rudang R, Zoulakis M, Wallander M, Darelid A, Lorentzon M (2017). Type 2 diabetes Mellitus is Associated with Better Bone Microarchitecture but Lower Bone Material Strength and poorer physical function in Elderly women: a Population-based study. J Bone Min Res.

[5] Ahmad OS, Leong A, Miller JA, Morris JA, Forgetta V, Mujammami M, Richards JB (2017). A Mendelian randomization study of the effect of Type-2 diabetes and glycemic traits on bone Mineral Density. J Bone Min Res.

[6] Liu J, Cao L, Qian YW, Chen ZX, Guo SF, Sun WQ, He ZR (2018). The association between risk of limb fracture and type 2 diabetes mellitus. Oncotarget.

[7] Mabilleau G, Bouvard B (2020). Update on: effects of anti-diabetic drugs on bone metabolism. Expert Rev Endocrinol Metabolism.

[8] Lupsa BC, Kibbey RG, Inzucchi SE. Ketones: the double-edged sword of SGLT2 inhibitors? Diabetologia. 2023;66(1):23–32. 10.1007/s00125-022-05815-1 36255460

[9] Blau JE, Taylor SI (2018). Adverse effects of SGLT2 inhibitors on bone health. Nat Rev Nephrol.

[10] Sterne JAC, Savovic J, Page MJ, Elbers RG, Blencowe NS, Boutron I, Cates CJ, Cheng HY, Corbett MS, Eldridge SM (2019). RoB 2: a revised tool for assessing risk of bias in randomised trials. BMJ.

[11] Bolinder J, Ljunggren O, Johansson L, Wilding J, Langkilde AM, Sjöström CD, Sugg J, Parikh S (2014). Dapagliflozin maintains glycaemic control while reducing weight and body fat mass over 2 years in patients with type 2 diabetes mellitus inadequately controlled on metformin. Diabetes Obes Metabolism.

[12] Bailey CJ, Villegas ECM, Woo V, Tang W, Ptaszynska A, List JF (2015). Efficacy and safety of dapagliflozin monotherapy in people with type 2 diabetes: a randomized double-blind placebo-controlled 102-week trial. Diabet Med.

[13] Kohan DE (2014). Long-term study of patients with type 2 diabetes and moderate renal impairment shows that dapagliflozin reduces weight and blood pressure but does not improve glycemic control. Kidney Int.

[14] Rosenstock J, Vico M, Wei L, Salsali A, List JF (2012). Effects of dapagliflozin, an SGLT2 inhibitor, on HbA(1c), body weight, and hypoglycemia risk in patients with type 2 diabetes inadequately controlled on pioglitazone monotherapy. Diabetes Care.

[15] Wilding JPH, Woo V, Soler NG, Pahor AP, Sugg J, Rohwedder K, Parikh S (2012). Long-term efficacy of dapagliflozin in patients with type 2 diabetes mellitus receiving high doses of insulin a randomized trial. Ann Intern Med.

[16] Araki E, Onishi Y, Asano M, Kim H, Yajima T (2017). Efficacy and safety of dapagliflozin over 1 year as add-on to insulin therapy in Japanese patients with type 2 diabetes: the DAISY (Dapagliflozin added to patients under InSulin therapY) trial. Diabetes Obes Metabolism.

[17] Schumm-Draeger PM, Burgess L, Korányi L, Hruba V, Hamer-Maansson JE, de Bruin TWA (2015). Twice-daily dapagliflozin co-administered with metformin in type 2 diabetes: a 16-week randomized, placebo-controlled clinical trial. Diabetes Obes Metabolism.

[18] Wilding JP, Ferrannini E, Fonseca VA, Wilpshaar W, Dhanjal P, Houzer A (2013). Efficacy and safety of ipragliflozin in patients with type 2 diabetes inadequately controlled on metformin: a dose-finding study. Diabetes Obes Metab.

[19] Lu CH, Min KW, Chuang LM, Kokubo S, Yoshida S, Cha BS (2016). Efficacy, safety, and tolerability of ipragliflozin in Asian patients with type 2 diabetes mellitus and inadequate glycemic control with metformin: results of a phase 3 randomized, placebo-controlled, double-blind, multicenter trial. J Diabetes Invest.

[20] Han KA, Chon S, Chung CH, Lim S, Lee KW, Baik S, Jung CH, Kim DS, Park KS, Yoon KH (2018). Efficacy and safety of ipragliflozin as an add-on therapy to sitagliptin and metformin in Korean patients with inadequately controlled type 2 diabetes mellitus: a randomized controlled trial. Diabetes Obes Metab.

[21] Fonseca VA, Ferrannini E, Wilding JP, Wilpshaar W, Dhanjal P, Ball G, Klasen S (2013). Active- and placebo-controlled dose-finding study to assess the efficacy, safety, and tolerability of multiple doses of ipragliflozin in patients with type 2 diabetes mellitus. J Diabetes Complicat.

[22] Min KW, Ku BJ, Lee JH, Kim MS, Ahn KJ, Lee MK, Kokubo S, Yoshida S, Cho HJ, Cha BS (2017). Addition of ipragliflozin to metformin treatment in Korean patients with type 2 diabetes mellitus: subgroup analysis of a phase 3 trial. Diabetes Metabolism J.

[23] Kashiwagi A, Kazuta K, Takinami Y, Yoshida S, Utsuno A, Nagase I (2015). Ipragliflozin improves glycemic control in Japanese patients with type 2 diabetes mellitus: the BRIGHTEN study. Diabetol Int.

[24] Gallo S, Raji A, Calle RA, Pong A, Meyer C (2020). The effects of ertugliflozin on β-cell function: pooled analysis from four phase 3 randomized controlled studies. Diabetes Obes Metabolism.

[25] Ji L, Han P, Liu Y, Yang G, Dieu Van NK, Vijapurkar U, Qiu R, Meininger G (2015). Canagliflozin in Asian patients with type 2 diabetes on metformin alone or metformin in combination with sulphonylurea. Diabetes Obes Metab.

[26] Bilezikian JP, Watts NB, Usiskin K, Polidori D, Fung A, Sullivan D, Rosenthal N (2016). Evaluation of bone Mineral density and bone biomarkers in patients with type 2 diabetes treated with canagliflozin. J Clin Endocrinol Metab.

[27] Yale JF, Bakris G, Cariou B, Yue D, David-Neto E, Xi L, Figueroa K, Wajs E, Usiskin K, Meininger G (2013). Efficacy and safety of canagliflozin in subjects with type 2 diabetes and chronic kidney disease. Diabetes Obes Metab.

[28] Rosenstock J, Aggarwal N, Polidori D, Zhao Y, Arbit D, Usiskin K, Capuano G, Canovatchel W (2012). Dose-ranging effects of canagliflozin, a sodium-glucose cotransporter 2 inhibitor, as add-on to metformin in subjects with type 2 diabetes. Diabetes Care.

[29] Rodbard HW, Seufert J, Aggarwal N, Cao A, Fung A, Pfeifer M, Alba M (2016). Efficacy and safety of titrated canagliflozin in patients with type 2 diabetes mellitus inadequately controlled on metformin and sitagliptin. Diabetes Obes Metab.

[30] Sha S, Polidori D, Heise T, Natarajan J, Farrell K, Wang SS, Sica D, Rothenberg P, Plum-Mörschel L (2014). Effect of the sodium glucose co-transporter 2 inhibitor canagliflozin on plasma volume in patients with type 2 diabetes mellitus. Diabetes Obes Metab.

[31] Sone H, Kaneko T, Shiki K, Tachibana Y, Pfarr E, Lee J, Tajima N (2020). Efficacy and safety of empagliflozin as add-on to insulin in Japanese patients with type 2 diabetes: a randomized, double-blind, placebo-controlled trial. Diabetes Obes Metab.

[32] Rau M, Thiele K, Hartmann NUK, Möllmann J, Wied S, Hohl M, Marx N, Lehrke M. Effects of empagliflozin on markers of calcium and phosphate homeostasis in patients with type 2 diabetes– data from a randomized, placebo-controlled study. Bone Rep. 2022;16. 10.1016/j.bonr.2022.101175 PMC8857445 35242892

[33] Kullmann S, Hummel J, Wagner R, Dannecker C, Vosseler A, Fritsche L, Veit R, Kantartzis K, Machann J, Birkenfeld AL (2022). Empagliflozin improves insulin sensitivity of the hypothalamus in humans with prediabetes: a Randomized, Double-Blind, Placebo-Controlled, phase 2 trial. Diabetes Care.

[34] Usiskin K, Kline I, Fung A, Mayer C, Meininger G (2014). Safety and tolerability of canagliflozin in patients with type 2 diabetes mellitus: pooled analysis of phase 3 study results. Postgrad Med.

[35] Kohler S, Zeller C, Iliev H, Kaspers S (2017). Safety and Tolerability of Empagliflozin in patients with type 2 diabetes: pooled analysis of phase I–III clinical trials. Adv Therapy.

[36] Brown JP, Don-Wauchope A, Douville P, Albert C, Vasikaran SD (2022). Current use of bone turnover markers in the management of osteoporosis. Clin Biochem.

[37] Greenblatt MB, Tsai JN, Wein MN (2017). Bone turnover markers in the diagnosis and monitoring of metabolic bone disease. Clin Chem.

[38] Dong B, Lv R, Wang J, Che L, Wang Z, Huai Z, Wang Y, Xu L (2022). The Extraglycemic Effect of SGLT-2is on Mineral and Bone metabolism and bone fracture. Front Endocrinol (Lausanne).

[39] Tu MY, Chen HL, Tung YT, Kao CC, Hu FC, Chen CM (2015). Short-term effects of Kefir-fermented milk consumption on bone Mineral density and bone metabolism in a Randomized Clinical Trial of osteoporotic patients. PLoS ONE.

[40] Shaffner J, Chen B, Malhotra DK, Dworkin LD, Gong R (2021). Therapeutic targeting of SGLT2: a new era in the treatment of Diabetes and Diabetic kidney disease. Front Endocrinol (Lausanne).

[41] Thrailkill KM, Nyman JS, Bunn RC, Uppuganti S, Thompson KL, Lumpkin CK, Kalaitzoglou E, Fowlkes JL (2017). The impact of SGLT2 inhibitors, compared with insulin, on diabetic bone disease in a mouse model of type 1 diabetes. Bone.

[42] Ye Y, Zhao C, Liang J, Yang Y, Yu M, Qu X. Effect of sodium-glucose co-transporter 2 inhibitors on bone metabolism and fracture risk. Front Pharmacol. 2019;9. 10.3389/fphar.2018.01517 PMC6331441 30670968

[43] Razzaque MS (2022). Interactions between FGF23 and vitamin D. Endocr Connections.

[44] Barsony J, Sugimura Y, Verbalis JG (2011). Osteoclast response to low Extracellular Sodium and the mechanism of hyponatremia-induced bone loss *. J Biol Chem.

[45] Watanabe-Takano H, Ochi H, Chiba A, Matsuo A, Kanai Y, Fukuhara S, Ito N, Sako K, Miyazaki T, Tainaka K (2021). Mechanical load regulates bone growth via periosteal osteocrin. Cell Rep.

[46] Schmitz D, Ek WE, Berggren E, Höglund J, Karlsson T, Johansson Å (2021). Genome-Wide Association Study of Estradiol Levels and the Causal Effect of Estradiol on Bone Mineral density. J Clin Endocrinol Metab.

[47] Schwartz AV, Johnson KC, Kahn SE, Shepherd JA, Nevitt MC, Peters AL, Walkup MP, Hodges A, Williams CC, Bray GA (2012). Effect of 1 year of an intentional weight loss intervention on bone mineral density in type 2 diabetes: results from the look AHEAD randomized trial. J bone Mineral Research: Official J Am Soc Bone Mineral Res.

[48] Watts NB, Bilezikian JP, Usiskin K, Edwards R, Desai M, Law G, Meininger G (2016). Effects of Canagliflozin on fracture risk in patients with type 2 diabetes Mellitus. J Clin Endocrinol Metabolism.

