# Supplementary material for: Effects of sodium-glucose cotransporter 2 inhibitors on bone metabolism in patients with type 2 diabetes mellitus: a systematic review and meta-analysis
Source: BMC Endocr Disord. 2024 Apr 24;24:52. doi: 10.1186/s12902-024-01575-8 (PMC11040974; doi:10.1186/s12902-024-01575-8)
Supplement: Supplementary file 1 — Supplementary Material 1 [file 12902_2024_1575_MOESM1_ESM.docx]

**Supplementary Table**

**Table S1** Search Strategy

| **Data source** | **Search terms** |
| --- | --- |
| **PubMed** | #1 (((((((((((((Sodium-Glucose Transporter 2 Inhibitors[MeSH Terms]) OR (Sodium Glucose Transporter 2 Inhibitors)) OR (SGLT-2 Inhibitors)) OR (SGLT 2 Inhibitors)) OR (SGLT2 Inhibitors)) OR (Sodium-Glucose Transporter 2 Inhibitor)) OR (Sodium Glucose Transporter 2 Inhibitor)) OR (SGLT2 Inhibitor)) OR (Inhibitor, SGLT2)) OR (Gliflozins)) OR (Gliflozin)) OR (SGLT-2 Inhibitor)) OR (Inhibitor, SGLT-2)) OR (SGLT 2 Inhibitor) |
|  | #2 (((((((Diabetes Mellitus, Type 2[MeSH Terms]) OR (diabetes mellitus type 2)) OR (type 2 diabetes mellitus)) OR (type 2 diabetes)) OR (T2D)) OR (T2DM)) OR (non-insulin-dependent diabetes mellitus)) OR (NIDDM) |
|  | #3 (Randomized Controlled Trial[Publication Type]) OR (controlled clinical trial[Publication Type]) |
|  | #4 #1 AND #2 AND #3 |
| **Web of Science** | TS=(Sodium-Glucose Transporter 2 Inhibitors OR Sodium Glucose Transporter 2 Inhibitors OR SGLT-2 Inhibitors OR SGLT 2 Inhibitors OR SGLT2 Inhibitors OR Sodium-Glucose Transporter 2 Inhibitor OR Sodium Glucose Transporter 2 Inhibitor OR SGLT2 Inhibitor OR Inhibitor, SGLT2 OR Gliflozins OR Gliflozin OR SGLT-2 Inhibitor OR Inhibitor, SGLT-2 OR SGLT 2 Inhibitor) AND TS=(Diabetes Mellitus, Type 2 OR diabetes mellitus type 2 OR type 2 diabetes mellitus OR type 2 diabetes OR T2D OR T2DM OR non-insulin-dependent diabetes mellitus OR NIDDM) AND TS=(Randomized Controlled Trial OR controlled clinical trial) |
| **Embase** | #1 'sodium glucose cotransporter 2 inhibitor'/exp OR 'sodium glucose cotransporter 2 inhibitor'  #2 'sodium glucose transporter 2 inhibitors':ab,kw,ti OR 'sglt-2 inhibitors':ab,kw,ti OR 'sglt 2 inhibitors':ab,kw,ti OR 'sglt2 inhibitors':ab,kw,ti OR 'sodium-glucose transporter 2 inhibitor':ab,kw,ti OR 'sodium glucose transporter 2 inhibitor':ab,kw,ti OR 'sglt2 inhibitor':ab,kw,ti OR 'inhibitor, sglt2':ab,kw,ti OR 'gliflozins':ab,kw,ti OR 'gliflozin':ab,kw,ti OR 'sglt-2 inhibitor':ab,kw,ti OR 'inhibitor, sglt-2':ab,kw,ti OR 'sglt 2 inhibitor':ab,kw,ti  #3 #1 OR #2  #4 'non insulin dependent diabetes mellitus'/exp OR 'non insulin dependent diabetes mellitus'  #5 'diabetes mellitus, type 2':ab,kw,ti OR 'diabetes mellitus type 2':ab,kw,ti OR 'type 2 diabetes mellitus':ab,kw,ti OR 'type 2 diabetes':ab,kw,ti OR 't2d':ab,kw,ti OR 't2dm':ab,kw,ti OR 'non-insulin-dependent diabetes mellitus':ab,kw,ti OR 'niddm':ab,kw,ti  #6 #4 OR #5  #7 'randomized controlled trial'/exp OR 'randomized controlled trial'  #8 #3 AND #6 AND #7 |
| **Cochrane databases** | #1 "SGLT2 Inhibitors" OR "SGLT-2 Inhibitors" OR "SGLT2 Inhibitor" OR "Gliflozins" OR "Sodium Glucose Transporter 2 Inhibitors" OR "SGLT 2 Inhibitors" OR "SGLT 2 Inhibitor" OR "SGLT2" OR "Gliflozin" OR "Sodium-Glucose Transporter 2 Inhibitor" OR "SGLT-2 Inhibitor" OR "Sodium Glucose Transporter 2 Inhibitor"  #2 MeSH descriptor: [Diabetes Mellitus, Type 2] explode all #1  #3 #1 AND #2 |
| **Scopus** | ( TITLE-ABS-KEY ( "sglt2 inhibitors*" OR "sglt-2 inhibitors*" OR "sglt2 inhibitor*" OR "gliflozins*" OR "sodium glucose transporter 2 inhibitors*" OR "sglt 2 inhibitors*" OR "sglt 2 inhibitor*" OR "sglt2*" OR "gliflozin*" OR "sodium-glucose transporter 2 inhibitor*" OR "sglt-2 inhibitor*" OR "sodium glucose transporter 2 inhibitor*" ) ) AND ( TITLE-ABS-KEY ( "Diabetes Mellitus, Type 2*" OR "diabetes mellitus type 2*" OR "type 2 diabetes mellitus*" OR "type 2 diabetes*" OR "T2D*" OR "T2DM*" OR "non-insulin-dependent diabetes mellitus*" OR "NIDDM*" ) ) AND ( TITLE-ABS-KEY ( "randomized controlled trial*" ) ) |

**Table S2** Results of publication bias test based on the Egger’s test

| Outcomes | Egger’s test (p-value) |
| --- | --- |
| BMD | 0.3993 |
| ALP | 0.5824 |
| PTH | 0.4211 |
| CTX | 0.5452 |
| P1NP | 0.9078 |
| Osteocalcin | 0.4647 |

Abbreviations: BMD, Bone mineral density; ALP, Alkaline phosphatase; PTH, Parathyroid hormone; CTX, Cross-linked C-terminal telopeptides of type I collagen; P1NP, Procollagen type 1 N-terminal propeptide
